# Knowledge, attitudes, and practices related to diabetic foot care among individuals with diabetes in India: a systematic review and meta-analysis

**DOI:** 10.1186/s12889-026-26295-7

**Published:** 2026-01-30

**Authors:** Bhukya Nom Kumar Naik, Mohd Jahid Riyaz Ahmad Khan, Sushma Prabhath, Debra Kerr, Arun G. Maiya, Anastasia Hutchinson, Elsa Sanatombi Devi, Bodil Rasmussen, Prabhath Matpady, Shubhada Karanth, Sahana Shetty

**Affiliations:** 1 Department of Anatomy, Kasturba Medical College Manipal, Manipal Academy of Higher Education (MAHE), Manipal, Karnataka 576104 India; 2 Faculty of Health/School of Nursing & Midwifery/Institute for Health Transformation, Deakin University, Geelong, VIC Australia; 3 Department of Forensic Medicine, Kasturba Medical College Manipal, Manipal Academy of Higher Education (MAHE), Manipal, Karnataka 576104 India; 4 Department of Anatomy and Medical Education, Kasturba Medical College Manipal, MAHE-FAIMER International Institute for Leadership in Interprofessional Education (M-FIILIPE)- FAIMER Regional Institute (FRI), Manipal Academy of Higher Education (MAHE), Manipal, Karnataka 576104 India; 5 School of Nursing & Midwifery, Centre for Quality & Patient Safety Research in the Institute for Health Transformation, Deakin University, Geelong, VIC Australia; 6 Department of Physiotherapy, Manipal College of Health Professions, Manipal, Manipal Academy of Higher Education, Manipal, Centre for Diabetic Foot Care and Research (CDFCR), Manipal, Karnataka 576104 India; 7 Department of Medical-Surgical Nursing, Manipal College of Nursing, Manipal, Manipal Academy of Higher Education (MAHE), Manipal, M-FIILIPE (FRI), Centre for Continuing Education and Interprofessional Development (CCEID), Manipal, Karnataka 576104 India; 8 WASH and CCES Policy Advocacy Network Expert (CiC), UNICEF - Hyderabad Field Office of Andhra Pradesh, Karnataka and Telangana, Telangana, Karnataka India; 9 Department of Medicine, Kasturba Medical College Manipal, Manipal Academy of Higher Education (MAHE), Manipal, Udupi, Karnataka 576104 India; 10 Department of Endocrinology, Kasturba Medical College Manipal, Manipal Academy of Higher Education (MAHE), Manipal, Udupi, Karnataka 576104 India

**Keywords:** Diabetic foot care, Knowledge, Attitude, Practice (KAP), Diabetes mellitus, Individuals with diabetes, India

## Abstract

**Background:**

Diabetic foot complications are a significant public health concern in India, leading to increased morbidity, amputations, and healthcare costs. Assessing the knowledge, attitudes, and practices (KAP) of individuals with diabetes toward foot care is essential for designing effective prevention and management strategies.

**Objective:**

This review aimed to synthesize evidence on diabetic foot care-related KAP among individuals with diabetes in India.

**Methods:**

A systematic search was conducted across major databases, grey literature sources, and reference lists of identified studies using predefined keywords up to May 16, 2025. Articles were screened on the basis of predefined inclusion criteria, and data from eligible studies were independently extracted. Pooled estimates were derived using a random-effects meta-analysis model, and the risk of bias was assessed using the Newcastle‒Ottawa Scale for cross-sectional studies (NOS-xs2).

**Results:**

Twenty studies published between 2012 and 2025, covering 13 Indian states and 2 Union territories, were included. The findings revealed low levels of good knowledge (32%), favourable attitudes (20%), and effective practices (26%) towards foot care. Although substantial heterogeneity was observed, minimal publication bias strengthened confidence in the results. Regional disparities highlighted significant underrepresentation from large parts of the country with diverse cultural and healthcare contexts.

**Conclusion:**

The review identified major gaps in diabetic foot care knowledge across India, with nearly two-thirds lacking patient-centred awareness or preventive behaviours. Addressing these challenges requires culturally tailored education and interprofessional strategies to reinforce preventive practices across healthcare. Regionally inclusive research and coordinated national initiatives are crucial to strengthening self-care, reducing complications, and easing the diabetic foot disease burden in India.

**Supplementary Information:**

The online version contains supplementary material available at 10.1186/s12889-026-26295-7.

## Background

Diabetic foot syndrome is a prevalent and severe complication of diabetes, leading to significant morbidity, reduced quality of life, and a substantial economic burden on both patients and society [1]. India is experiencing a rapid increase in diabetes incidence, with 69.2 million people affected in 2015 (8.8% prevalence), and projections indicating that this number could reach 123.5 million by 2040, making India the potential diabetes capital of the world [2–5]. According to the latest IDF Diabetes Atlas (11th edition, 2025), as of 2024, 19.3 million people in India are living with diabetes, and this number is projected to rise to 45.8 million by 2050 [6]. Among adults aged 20–79 years, nearly 43.0% (approximately 38.6 million) remain undiagnosed, highlighting that India accounts for 1 in 7 adults with diabetes globally [6]. As of 2024, currently 90.0 million adults aged 20–79 years are living with diabetes, and it is projected to reach 157.0 million by 2050. In Southeast Asia (India, Bangladesh, Sri Lanka, Nepal and Mauritius) [6], the number of adults living with diabetes is predicted to increase by 73% to 185 million by 2050 [6].

Diabetic foot complications continue to be a critical public health concern, largely driven by inadequate knowledge, suboptimal foot care attitudes, and poor self-care practices among people living with diabetes [7]. This situation is further compounded by limited awareness [8–11], restricted access to foot care services [9, 11, 12], and delayed health-seeking behaviors [10, 11], underscoring the urgent need for targeted educational and preventive interventions.

Foot care plays a vital role in preventing ulcers, infections, and amputations. However, it is often overlooked in diabetes management at both the individual and system levels. Timely identification of foot problems, regular inspection, and adherence to appropriate self-care practices can significantly reduce complications [13]. Despite this, these preventive measures are not widely adopted in routine self-care among individuals with diabetes in India, as evidenced by several studies [14–18].

Foot complications frequently result from neuropathy [19–21], poor circulation [22], and unnoticed minor injuries that progress due to neglect [20, 23]. These risks can be minimized through consistent foot care practices, which are closely linked to individuals’ knowledge and attitudes towards foot health. Unfortunately, studies across different parts of India have highlighted widespread gaps in understanding and adhering to recommended foot care practices [24–26]. This variation is often influenced by sociodemographic factors such as age, education, income, and previous foot-related experiences [1, 24, 26].

Diabetes-related amputations are among the most serious and costly outcomes of diabetic foot complications. Globally, 40–70% of all lower extremity amputations (LEAs) are attributed to diabetes, 85% of which are preceded by foot ulcers [27, 28]. The global incidence rate of minor amputations is approximately 139.97 per 100,000 individuals with diabetes, while major amputations occur at a rate of 94.82 per 100,000 individuals [29]. The 5-year mortality rate following major amputation can range from 50 to 80% [30]. Alarmingly, individuals with diabetes are 25 times more likely to undergo leg amputations than nondiabetic individuals are [28].

The prevalence of amputation among individuals with type 2 diabetes in India was reported to be 3% in one multicentric study by Tiwari et al., 2014 [31], whereas another study reported an overall amputation rate of 28.4% among patients with DFU [32]. The mortality and recurrence of ulcers also remain significant concerns [33].

The potential benefits of structured diabetic foot education and preventive strategies have been previously highlighted by multiple studies and reviews [34–36]. A global meta-analysis revealed that foot care education improved knowledge and behavior, although changes in self-efficacy were modest [27]. Clinical guidelines consistently recommend early foot evaluations, debridement, and therapeutic footwear as essential components of care [28]. However, despite these findings, no single intervention has been proven to be universally effective in reducing the incidence of ulcers and the number of amputations without long-term implementation [28]. This emphasizes the need for integrated, long-term strategies, particularly in resource-constrained settings such as India.

The lack of a national-level synthesis of KAP data has hampered the formulation of unified and targeted policy recommendations to improve diabetic foot care practices at a larger scale. Regional disparities further complicate the picture. For example, a recent meta-analysis estimated that the national pooled prevalence of diabetic foot ulcers (DFUs) among individuals with diabetes is 6.2%, with regional variations: 9.5% in East India, 7.4% in South India, and 5.6% in North India [37]. Hospital-based studies reported a higher DFU incidence (7.5%) than community-based studies did (2.5%) [37]. Factors contributing to an increased risk of DFUs include longer diabetes duration, male sex, older age, comorbidities, and poor lifestyle choices [37].

These findings highlight the urgent need for a comprehensive national review. Understanding the current levels of KAP surrounding diabetic foot care in India is critical for designing appropriate interventions. Hence, this systematic review and meta-analysis aims to evaluate and summarize existing data on foot care KAP among people with diabetes in India, compare regional and setting-based differences, identify associated risk factors, and guide evidence-based foot care education strategies.

## Methods

### Search strategies

A comprehensive search strategy was developed in accordance with the PRISMA 2020 guidelines and checklist [38] to identify studies assessing KAPs related to diabetic foot care among individuals with diabetes in India. Two independent reviewers (BNKN and MJRAK) conducted the search across eight major electronic databases: PubMed (*n* = 145), Embase (*n* = 48), Scopus (*n* = 1,516), LILACS (*n* = 125), Cochrane Library (*n* = 28), ProQuest (*n* = 14), Web of Science (*n* = 18), and CINAHL Ultimate (*n* = 0), covering all records published up to May 16, 2025. Keywords and Medical Subject Headings (MeSH) such as "diabetic foot," "self-care," "diabetes mellitus," and "health knowledge, attitudes, and practice" were combined via Boolean operators (AND/OR). The search strings are presented in Table 1.

**Table 1:** Search strings and results from multiple databases (PubMed, Scopus, Embase, LILACS, Cochrane, ProQuest, CINAHL, and Web of Science)

**Database**	**Search Key Words**	**Number of results**	**Results**
PubMed	(((Diabetic Foot) AND (Self Care)) AND (Health Knowledge, Attitudes, Practice)) AND (Diabetes Mellitus) ("diabetic foot"[MeSH Terms] OR ("diabetic"[All Fields] AND "foot"[All Fields]) OR "diabetic foot"[All Fields]) AND ("self care"[MeSH Terms] OR ("self"[All Fields] AND "care"[All Fields]) OR "self care"[All Fields]) AND ("health knowledge, attitudes, practice"[MeSH Terms] OR ("health"[All Fields] AND "knowledge"[All Fields] AND "attitudes"[All Fields] AND "practice"[All Fields]) OR "practice attitudes health knowledge"[All Fields] OR "health knowledge attitudes practice"[All Fields]) AND ("diabetes mellitus"[MeSH Terms] OR ("diabetes"[All Fields] AND "mellitus"[All Fields]) OR "diabetes mellitus"[All Fields]) Diabetic Foot: "diabetic foot"[MeSH Terms] OR ("diabetic"[All Fields] AND "foot"[All Fields]) OR "diabetic foot"[All Fields] Self Care: "self care"[MeSH Terms] OR ("self"[All Fields] AND "care"[All Fields]) OR "self care"[All Fields] Health Knowledge, Attitudes, Practice: "health knowledge, attitudes, practice"[MeSH Terms] OR ("health"[All Fields] AND "knowledge"[All Fields] AND "attitudes"[All Fields] AND "practice"[All Fields]) OR "practice attitudes health knowledge"[All Fields] OR "health knowledge, attitudes, practice"[All Fields] Diabetes Mellitus: "diabetes mellitus"[MeSH Terms] OR ("diabetes"[All Fields] AND "mellitus"[All Fields]) OR "diabetes mellitus"[All Fields]	145	Inception to 16–05–2025
Scopus	((ALL ((diabetic AND foot)) AND ALL ((self AND care)) AND ALL ((health AND knowledge, AND attitudes, AND practice)) AND ALL ((diabetes AND mellitus)))	1516	Inception to 16–05–2025
Embase	(‘diabetic foot’/exp OR ‘diabetic foot’) AND ‘self care’ AND ‘attitude to health’ AND ‘diabetes mellitus’	48	Inception to 16–05–2025
LILACS (VHL)	(Diabetic Foot) AND (Self Care) AND (Health Knowledge, Attitudes, Practice) AND (Diabetes Mellitus)	110	Inception to 16–05–2025
LILACS (Plus)	(Diabetic Foot) AND (Self Care) AND (Health Knowledge, Attitudes, Practice) AND (Diabetes Mellitus)	15	Inception to 16–05–2025
Cochrane	IDSearch #1Diabetic Foot #2Self Care #3Health Knowledge, Attitudes, Practice #4Diabetes Mellitus #5#1 AND #2 AND #3 AND #4	28	Inception to 16–05–2025
ProQuest	noft(foot diabetic) AND noft(Self Care) AND noft(Health Knowledge, Attitudes, Practice) AND noft(Diabetes Mellitus)	14	Inception to 16–05–2025
CINAHL Ultimate	diabetic foot AND (self care or self-care or self-management or self management) AND health knowledge, attitudes, practice AND (diabetes mellitus or diabetes or diabetic)	0	Inception to 16–05–2025
Web of Science	Diabetic Foot (All Fields) AND Self Care (All Fields) AND Health Knowledge, Attitudes, Practice (All Fields) AND Diabetes Mellitus (All Fields)	18	Inception to 16–05–2025
Total		**1,894**	

### Eligibility criteria

Articles were included if they were full-text, peer-reviewed, or gray literature studies published in English, conducted within India, and available up to May 2025. Eligible study designs included observational, cross-sectional, cohort, and case‒control studies conducted on human participants aged 18 years and above. Only studies reporting KAP related to diabetic foot care among individuals with type 1 or type 2 diabetes, regardless of the presence of diabetic foot ulcers, from either rural or urban areas were considered. Only preintervention data were included in this review to provide an unbiased representation of baseline KAP toward diabetic foot care. Studies were excluded if they were conducted outside India, involved nondiabetic populations, lacked accessible full text despite exhaustive retrieval attempts, or failed to report quantitative data on KAP.

### Outcome measures

The current study aimed to provide pooled data from the included studies to assess the levels of knowledge, attitudes, and practices related to diabetic foot care across Indian settings. It evaluated the proportions of participants with poor, satisfactory, and good knowledge; favourable and unfavourable attitudes; and poor, satisfactory, and good (effective) practices based on the scoring systems and criteria reported in the studies.

### Data extraction

**Fig. 1:**
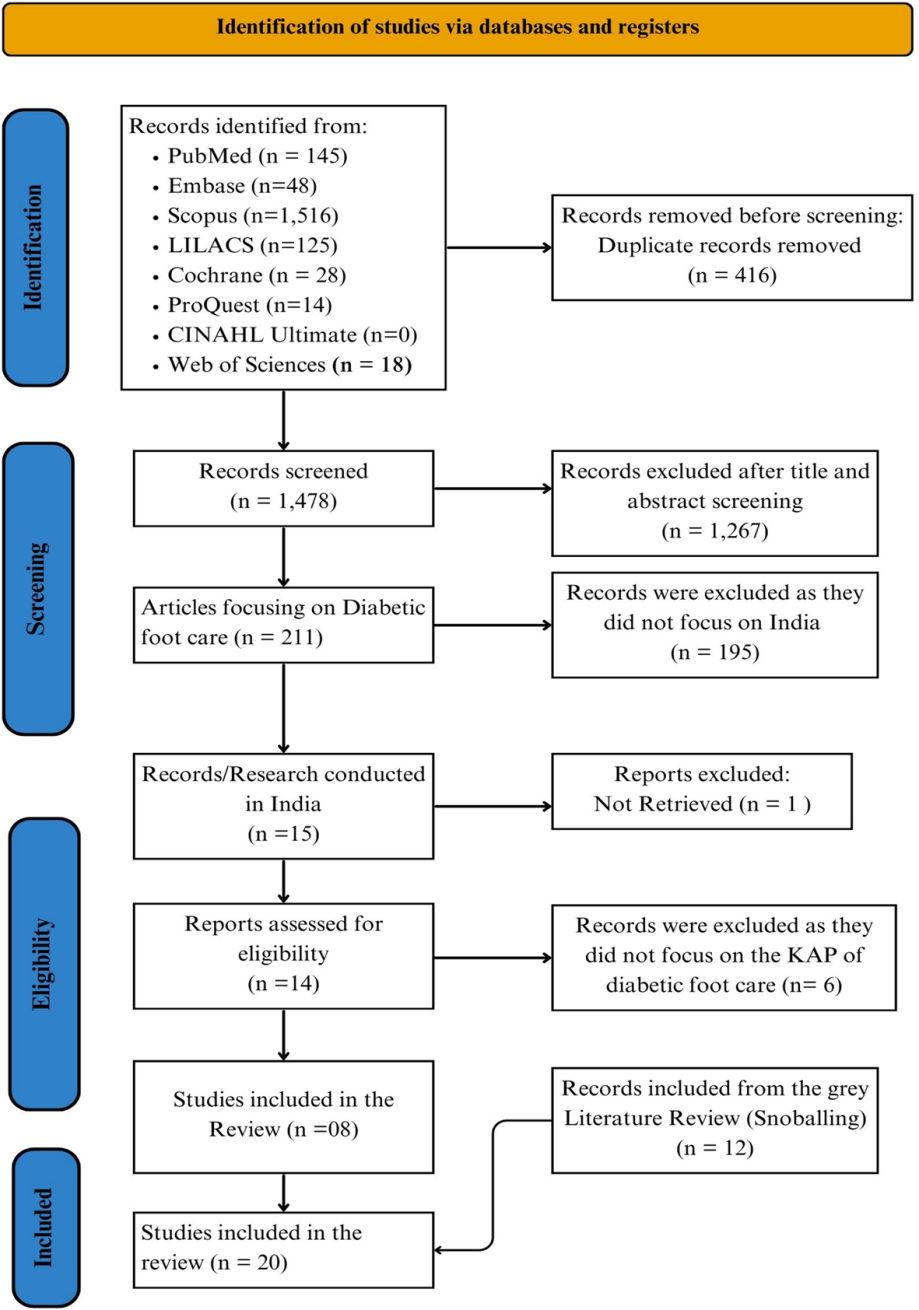
PRISMA flow diagram of the literature selection process

Data were extracted via the standardized JBI format, including the first author’s name, year of publication, study design, sample size, and proportion of participants with varying levels of knowledge (poor, satisfactory, good)**,** attitudes (favourable, unfavourable), and practices (poor, satisfactory, good) related to diabetic foot care. Following two-stage screening and inclusion of additional studies from gray literature, 20 studies were ultimately included, as illustrated in the PRISMA flow diagram (Fig. 1). Any discrepancies were resolved, when necessary, through discussion with a third reviewer (SP).

The reported percentages were first converted into the actual number of cases via a standard formula. This approach is particularly helpful in research when only the percentage of participants with a specific attribute (e.g., good knowledge) is provided, and the corresponding number of individuals must be estimated. The formula used is as follows:

$$Number\ of \ cases=\left(\frac{Percentage }{100}\right)\times Sample\ Size$$

For example, if a study reported that 60% of participants had good knowledge out of a total sample size of 150, the calculation would be as follows:

$$Number\ of\ cases=\left(\frac{60 }{100}\right)\times 150=90$$

This means that 90 participants had good knowledge.

Since the calculation yielded a decimal, we round to the nearest whole number to ensure a realistic representation of individual counts.

These values were then used to construct forest plots via a random-effects model with Freeman–Tukey double arcsine transformation to stabilize variance and address heterogeneity among the included studies.

### Risk of bias assessment

The methodological quality of the included studies was assessed via the Newcastle‒Ottawa Scale for cross-sectional studies (NOS-xs2) [39], which evaluates three main domains: representativeness of the sample, justification of sample size, and reliability of outcome measurement. A maximum of four stars could be awarded: one for each of the first two domains and up to two for the outcome assessment. Studies receiving 3–4 stars were considered low risk, those with 2 stars were considered moderate risk, and those scoring 0–1 stars were considered high risk. Two reviewers (BNKN and SP) independently conducted the risk of bias assessments, with any disagreements settled through discussion or by involving a third reviewer (MJRAK).

### Data processing and analysis

All the extracted data were compiled in Microsoft Excel and analysed via the web software Meta-analysis online (metaanalysisonline.com; supported by ELIXIR Hungary) [40]. This tool was chosen over standard statistical software such as R due to its validated implementation of proportion-based meta-analysis methods, ease of use, and suitability for handling prevalence data without requiring advanced programming expertise, thereby ensuring analytical transparency and reproducibility. The proportions of participants reporting knowledge (poor, satisfactory, and good), attitudes (Favourable and unfavourable), and practices (poor, satisfactory, and good) were computed for each study. To account for between-study heterogeneity, a random-effects model using the Freeman–Tukey double arcsine transformation was applied. The online platform implements standard meta-analytic algorithms comparable to those used in conventional statistical software, including inverse-variance weighting under a random-effects framework. Pooled prevalence estimates with 95% confidence intervals were generated, and forest plots were created to display the overall estimates and variability across studies. Heterogeneity was quantified via the I^2^ statistic, with values above 75% indicating substantial variability. Funnel plots, along with Egger’s regression and Begg’s correlation tests, were used to assess the presence of small-study effects or publication bias.

## Results

### Study identification

The database search and desk review yielded 1,894 articles from the identified electronic sources. After removing 416 duplicates, 1,478 records were screened via Rayyan software [41] by reviewers (BNKN and MJRAK), with 1,267 records excluded on the basis of title and abstract. In the second stage, 211 full-text articles were reviewed, and 196 were excluded because they were not published in Indian settings. Of the remaining 15 studies, one could not be retrieved, and six did not report the outcomes of interest (i.e., proportions of adequate knowledge, good attitudes, and effective preventive practices), leaving seven eligible studies from the database searches. An additional 12 studies were identified through gray literature using snowballing, resulting in a total of 20 included studies [13, 15, 42–59].

### Characteristics of the included studies

**Fig. 2:**
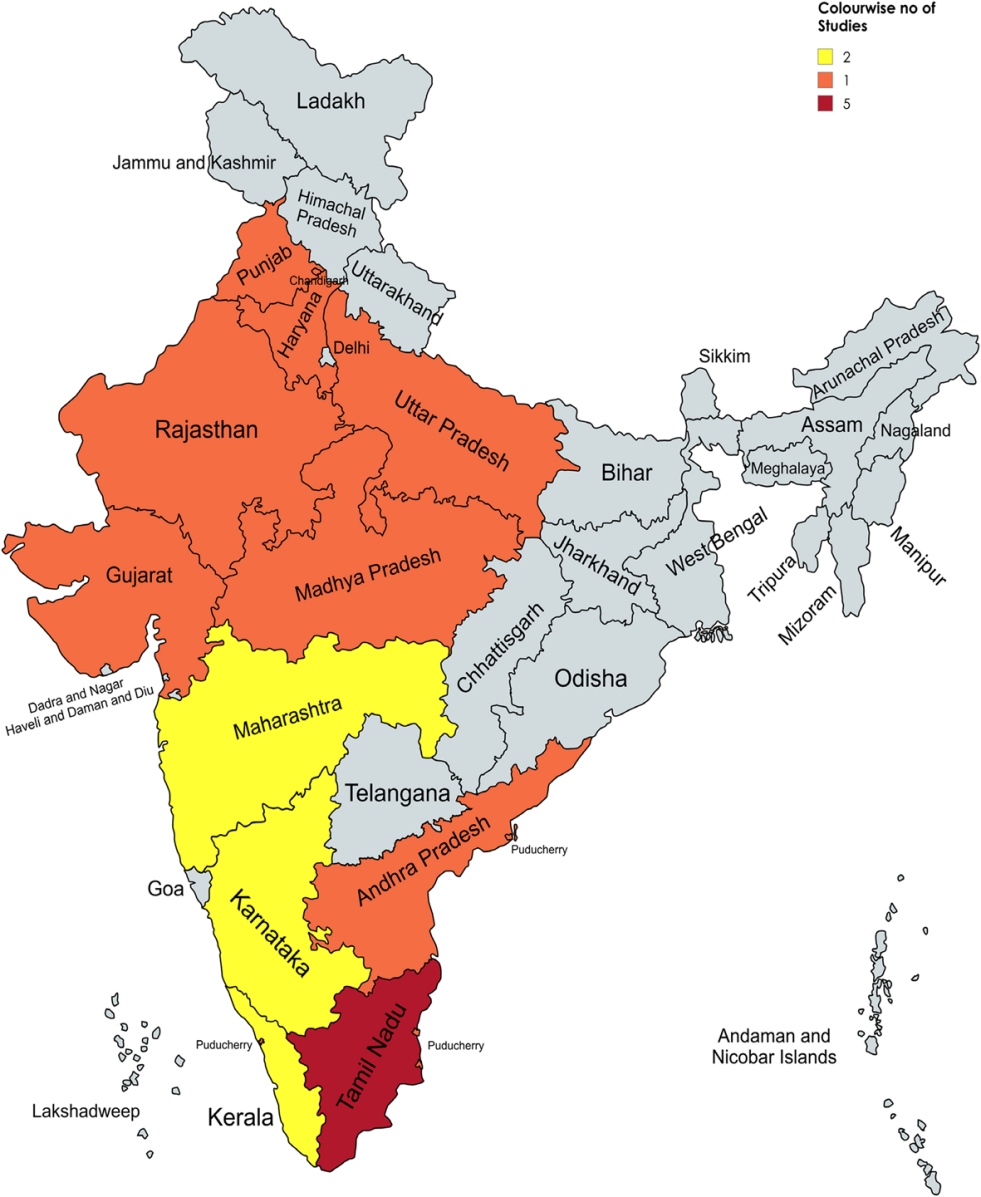
A coloured map of India illustrating the states and union territories where the included studies were conducted

A total of 20 studies representing diverse regions across 13 Indian states and 2 Union territories were included. The highest number of studies were from Tamil Nadu (5), followed by Kerala (2), Karnataka (2), and Puducherry (1), with others from Punjab (1), Maharashtra (2), Uttar Pradesh (1), and other states (Fig. 2**—**Generated with the help of (https://www.mapchart.net/) [60]. Most of the included studies used a cross-sectional observational design, and were either hospital- or community-based. Studies from Kochi [42], Lucknow [45], and Puducherry [54] provided separate data for DFU vs non-DFU and urban vs rural populations, which were extracted and analysed independently. The study populations primarily consisted of individuals with type 2 diabetes, although a few studies focused on high-risk groups, such as people with peripheral neuropathy and peripheral artery disease (PAD). The time span of the included studies ranged from 2012–2025, with a noticeable increase in publications after 2020, reflecting the growing attention given to diabetic foot complications in India. The key characteristics and reported findings from these studies are summarized in Table 2.

**Table 2:** Summary of the main characteristics and key findings of the included studies (As extracted using standardised JBI format)

Sl.No	Authors Name	Location	State	Study Design	Questionnaire Used	Population	Year of publication	Sample Size	Percentage								
									**Poor Knowledge**	**Satisfactory Knowledge**	**Good Knowledge**	**Unfavourable Attitude**	**Favourable Attitude**	**Good Attitude**	**Poor Practices**	**Satisfactory Practices**	**Good Practices**
1	Chellan et al. (DFU) [42]	Kochi	Kerala	Cross-sectional	Structured, validated KAP	Patients with DFU and without DFU (Type 2 DM)	2012	103	30.1	12.6	94.2	5.8	94.2	NA	39.8	43.5	16.7
2	Chellan et al. (No DFU) [42]	Kochi	Kerala	Cross-sectional	Structured, validated KAP	Diabetic clients attending outpatient department	2012	100	14	46	40	12	88	NA	9	55	36
3	Cecyli & Thamupriyadharshini [43]	Saveetha Medical College and Hospital, Chennai	Tamil Nadu	Descriptive cross-sectional	Self-structured	Diabetic clients attending outpatient department	2020	100	61	28	11	NA	NA	NA	77	0	23
4	Kumar Gupta et al. [15]	District Fatehgarh Sahib	Punjab	Community cross-sectional	NAFF (Nottingham, adapted)	Adults with Type 2 Diabetes in a rural area	2022	700	NA	NA	NA	NA	NA	NA	84	16	NA
5	Dhandapani et al. [13]	Rural field practice area of J.N. Medical College, Belagavi	Karnataka	Cross-sectional observational	Pretested (Hasnain et al.)	Type 2 diabetic patients attending hospital	2022	461	37.31	47.9	14.8	NA	NA	NA	19.5	63.6	16.9
6	Francis et al. [44]	Jodhpur	Rajasthan	Descriptive	Self-structured	Diabetic patients from a tertiary care hospital	2024	100	49	24	27	23	31	46	39	33	28
7	Kumar et al. (DFU) [45]	SGPGIMS, Lucknow (Tertiary care hospital)	Uttar Pradesh	Comparative cross-sectional	Structured (literature-based)	Patients with and without foot ulcer (Type 2 DM) –- With DFU	2015	200	52.3	NA	47.7	NA	NA	NA	NA	NA	NA
8	Kumar et al. (No DFU) [45]	SGPGIMS, Lucknow (Tertiary care hospital)	Uttar Pradesh	Comparative cross-sectional	Structured (literature-based)	Patients with and without foot ulcer (Type 2 DM) –- With out DFU	2015	200	48	NA	52	NA	NA	NA	NA	NA	NA
9	Gholap & Mohite [46]	Krishna Hospital, Karad	Maharashtra	Descriptive	Structured	Type 2 diabetic patients attending OPD	2019	50	18	58	24	NA	NA	NA	20	58	22
10	Sutariya & Kharadi [47]	Ahmedabad city	Gujarat	Cross-sectional, hospital-based	Based on Desalu et al	Patients with diabetes mellitus	2016	103	27	50	23	NA	NA	NA	51	33	16
11	Priyadharishini et al. [48]	CHAD and RUHSA (Secondary hospitals)	Tamil Nadu	Descriptive cross-sectional	Investigator-developed	Diabetic patients from urban and rural settings	2020	100	32	68	NA	NA	NA	NA	12	88	NA
12	Mani [49]	Kanchipuram district	Tamil Nadu	Descriptive cross-sectional	Structured, author-made	Diabetic patients attending diabetic clinic	2022	120	92	27	1	NA	NA	NA	64	34	2
13	Nagar et al. [50]	Tertiary care hospital, Bhopal	Madhya Pradesh	Hospital-based cross-sectional	Pretested, semi structured	Diabetic patients attending hospital in Bhopal	2018	150	34.6	49.3	16	56.6	43.3	NA	66	NA	34
14	Maniktalla et al. [51]	Pune	Maharashtra	Hospital-based cross-sectional	Validated, translated, 11 + 12 Qs	Type 2 diabetic patients from Pune	2025	165	95.7	4.25	NA	NA	NA	NA	90	10	NA
15	JV Jeevitha et al. [52]	Kovai Medical Centre & Hospital (KMCH), Coimbatore	Tamil Nadu	Descriptive, hospital-based	Validated foot care & risk tool	Patients with Type 2 diabetes at KMCH	2025	60	3	25	72	NA	NA	NA	10	88	1.6
16	Mehmi et al. [53]	Government Medical College & Hospital, Sector 32, Chandigarh	Chandigarh (UT)	Descriptive, tertiary hospital	Self-structured, expert validated	Diabetic patients visiting outpatient clinic	2021	100	18	78	4	14	86	NA	11	NA	89
17	Dheepa et al. (R) [54]	Puducherry	Puducherry (UT)	Comparative descriptive	Structured	Diabetic patients from rural and urban settings	2020	30	33.33	50	16.66	56.66	43.33	NA	53.33	NA	46.66
18	Dheepa et al. (U) [54]	Puducherry	Puducherry (UT)	Comparative descriptive	Structured	Diabetic patients from rural and urban settings	2020	30	10	56.66	33.33	26.66	73.33	NA	33.33	NA	66.66
19	Sivan et al. [55]	SK Hospital, Thiruvananthapuram	Kerala	Prospective with intervention	Structured, validated, 24 Qs	High-risk diabetic patients for foot ulcers	2021	100	65	29	6	1	28	71	87	12	1
20	Chandrakala et al. [56]	Tirupati	Andhra Pradesh	Descriptive, hospital-based	28-item structured checklist	Patients with Type 2 Diabetes attending SVIMS	2017	100	27	44	29	NA	NA	NA	NA	NA	NA
21	Meghana et al. [57]	SSMC, Tumkur and Karnataka Institute of Endocrinology and Research, Bangalore	Karnataka	Cross-sectional	Validated/adapted + Nottingham	Diabetic patients visiting outpatient clinic	2020	134	2.23	24.62	73.13	NA	NA	NA	4.47	73.13	22.38
22	Amit et al. [58]	CMC Vellore	Tamil Nadu	Cross-sectional outpatients	Nottingham Assessment 2015	Diabetic patients from Karnataka hospitals	2023	204	69	0	30.39	NA	NA	NA	NA	NA	NA
23	Verma et al. [59]	Haryana	Haryana	Community-based cross-sectional	Adapted/translated validated international	Rural population with Type 2 diabetes	2021	416	24	12.5	63.5	NA	NA	NA	20.6	32.7	46.7

### Risk of bias assessment of included studies

The quality of each study included in this review was assessed using the Newcastle–Ottawa Scale for cross-sectional studies (NOS-xs2). Among the studies, 13 (p65%) were rated as having a low risk of bias, 7 (35%) as having a moderate risk of bias, and none (0%) as having a high risk of bias (Table 3)*.*

**Table 3:** Bias assessment of the included studies (Using Newcastle–Ottawa Scale for cross-sectional studies (NOS-xs2))

Sl.No	Author's Name (Year)	STUDY SAMPLE SELECTION						ASSESSMENT OF OUTCOME				Total (★/4)	Risk of Bias
		1. Representativeness of the study sample (★)				2. Sample size (★)		3. Assessment of the outcome(s) (★★)					
		a) Truly representative (random sampling) (★)	b) Somewhat representative (non-random) (★)	c) Not representative (selected group) (-)	d) No description (-)	a) Justified and satisfactory (★)	b) Not justified or unsatisfactory (-)	a) Gold-standard tool (★★)	b) Acceptable tool (★)	c) Unacceptable method (-)	d) No description (-)		
1	Chellan et al., (2012) [42]	-	★	-	-	★	-	★★	-	-	-	★★★★	Low Risk
2	Cecyli & Thamupriyadharshini., (2020) [43]	-	★	-	-	-	-	-	★	-	-	★★	Moderate Risk
3	Kumar Gupta et al., (2022) [15]	-	★	-	-		-	★★	-	-	-	★★★	Low Risk
4	Dhandapani et al., (2022) [13]	-	★	-	-	★	-	★★	-	-	-	★★★★	Low Risk
5	Francis et al., (2024) [44]	-	★	-	-	-	-	-	★	-	-	★★	Moderate Risk
6	Kumar et al. (2015) [45]	-	★	-	-	★	-	-	★	-	-	★★★	Low Risk
7	Gholap & Mohite (2019) [46]	-	★	-	-	-	-	-	★	-	-	★★	Moderate Risk
8	Sutariya & Kharadi (2016) [47]	-	★	-	-	-	-	-	★	-	-	★★	Moderate Risk
9	Priyadharishini et al., (2020) [48]	-	★	-	-	★	-	-	★	-	-	★★★	Low Risk
10	Mani (2022) [49]	-	★	-	-	-	-	-	★	-	-	★★	Moderate Risk
11	Nagar et al., (2018) [50]	-	★	-	-	★	-	-	★★	-	-	★★★★	Low Risk
12	Maniktalla et al., (2025) [51]	-	★	-	-	★	-	-	★★	-	-	★★★★	Low Risk
13	JV Jeevitha et al., (2025) [52]	-	★	-	-	-	-	-	★	-	-	★★	Moderate Risk
14	Mehmi et al., (2021) [53]	-	★	-	-	★	-	-	★	-	-	★★★	Low Risk
15	Dheepa et al., (2020) [54]	-	★	-	-	-	-	-	★	-	-	★★	Moderate Risk
16	Sivan et al., (2021) [55]	-	★	-	-	★	-	-	★	-	-	★★★	Low Risk
17	Chandrakala et al., (2017) [56]	-	★	-	-	★	-	-	★	-	-	★★★	Low Risk
18	Meghana et al., (2020) [57]	-	★	-	-	★	-	★★	-	-	-	★★★★	Low Risk
19	Amit et al., (2023) [58]	-	★	-	-	★	-	★★	-	-	-	★★★★	Low Risk
20	Verma et al., (2021) [59]	-	★	-	-	★	-	★★	-	-	-	★★★★	Low Risk

#### Knowledge, Attitudes, and Practices (KAPs) towards diabetic foot care in India: a meta-analytic synthesis

A total of 20 studies were included in the review; however, data were extracted separately for each subdomain (e.g., knowledge: poor, satisfactory, and good; attitude: favourable and unfavourable; practice: poor, satisfactory, and good). Not all included studies reported data for every subcategory (for example, some studies did not report values for “good” knowledge). Consequently, the number of studies contributing to specific forest plot analyses differed across sublevels. Analysis was performed for the matching studies accordingly.

### Knowledge domain

#### Poor knowledge

A total of 17 studies involving 2,861 individuals were included in the meta-analysis. Individuals with or without DFU and individuals with diabetes from both rural and urban areas were included. The analysis was conducted to determine the proportion of participants with poor knowledge about diabetic foot care across various regions in India. The combined analysis revealed that 34% (95% CI: 24.0%–45.0%) had poor knowledge levels (Fig. 3.1A), with considerable heterogeneity (I^2^ = 97%, *p* < 0.01) and no publication bias (intercept: −1.98, 95% −9.81—5.85, t: −0.496, *p* value: 0.626); this finding was further corroborated by a symmetrical appearance in the funnel plot (Fig. 3.1B).

**Fig. 3:**
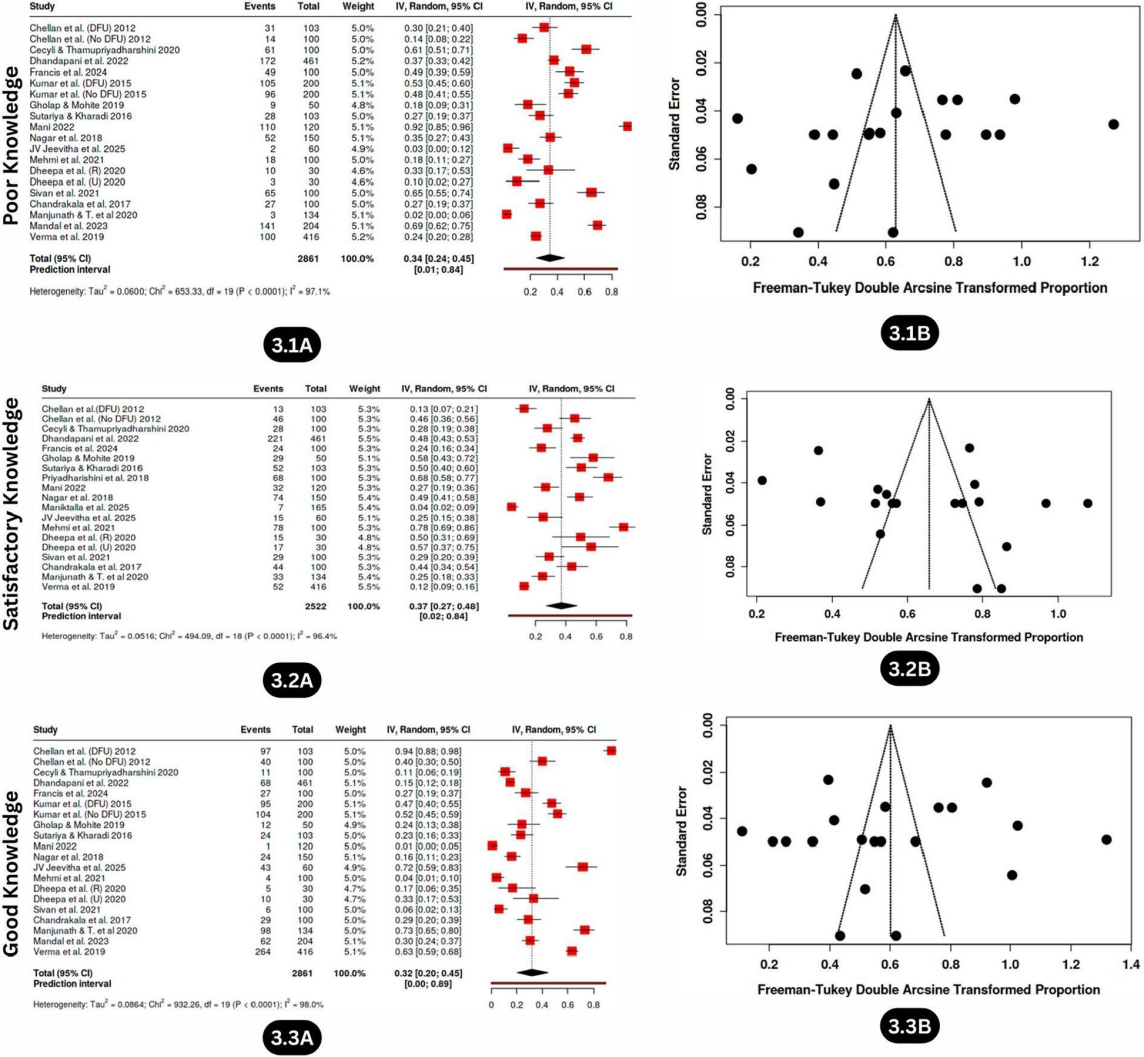
Forest plot and Funnel plot depicting the pooled estimates, publication bias of poor knowledge (3.1A, 3.1B), satisfactory knowledge (3.2A, 3.2B), and good knowledge (3.3A, 3.3B) levels related to diabetic foot care among individuals with diabetes in India

#### Satisfactory knowledge

In the 17 studies covering 2,522 individuals, the estimated proportion of individuals with satisfactory knowledge was 37% (95% CI: 27.0%–48.0%) (Fig. 3.2A), with high heterogeneity (I^2^ = 96%, *p* < 0.01), and no substantial publication bias was detected in either the funnel plot analysis (Fig. 3.2B) or Egger’s test (intercept: 3.85, 95% CI: −2.99–10.69, t: 1.104, *p* value: 0.285), suggesting variability in educational access and delivery.

#### Good knowledge

The same 17 studies involving 2,861 individuals also provided insights into good knowledge levels, with a pooled estimate of 32% (95% CI: 20.0%—45.0%) (Fig. 3.3A). Heterogeneity (I^2^ = 98%, *p* < 0.01) and no publication bias were confirmed by the funnel plot (Fig. 3.3B) and Egger’s regression (intercept: −1.94, 95% −11.31—7.43, t: −0.405, p value: 0.69), reflecting differing educational outcomes across populations.

### Attitude domain

#### Unfavourable attitude

Unfavourable attitudes toward diabetic foot care were assessed across six studies involving 713 participants from both rural and urban settings, including individuals with diabetes with and without diabetic foot ulcers. The pooled estimate showed that 21% (95% CI: 7.0%–42.0%) of participants held unfavourable attitudes toward diabetic foot care (Fig. 4.1A). Substantial heterogeneity was observed (I^2^ = 96%, *p* < 0.01; Q = 182.90, df = 7), indicating marked variability across studies. Although publication bias was explored using funnel plot inspection and Egger’s regression test (intercept = 0.6, 95% CI: − 15.57–16.77; t = 0.073; *p* = 0.944), these analyses were conducted on an exploratory basis due to the limited number of included studies and were interpreted with caution. The absence of statistically significant small-study effects does not preclude bias but suggests no strong evidence of publication bias within the available data (Fig. 4.1B).

**Fig. 4:**
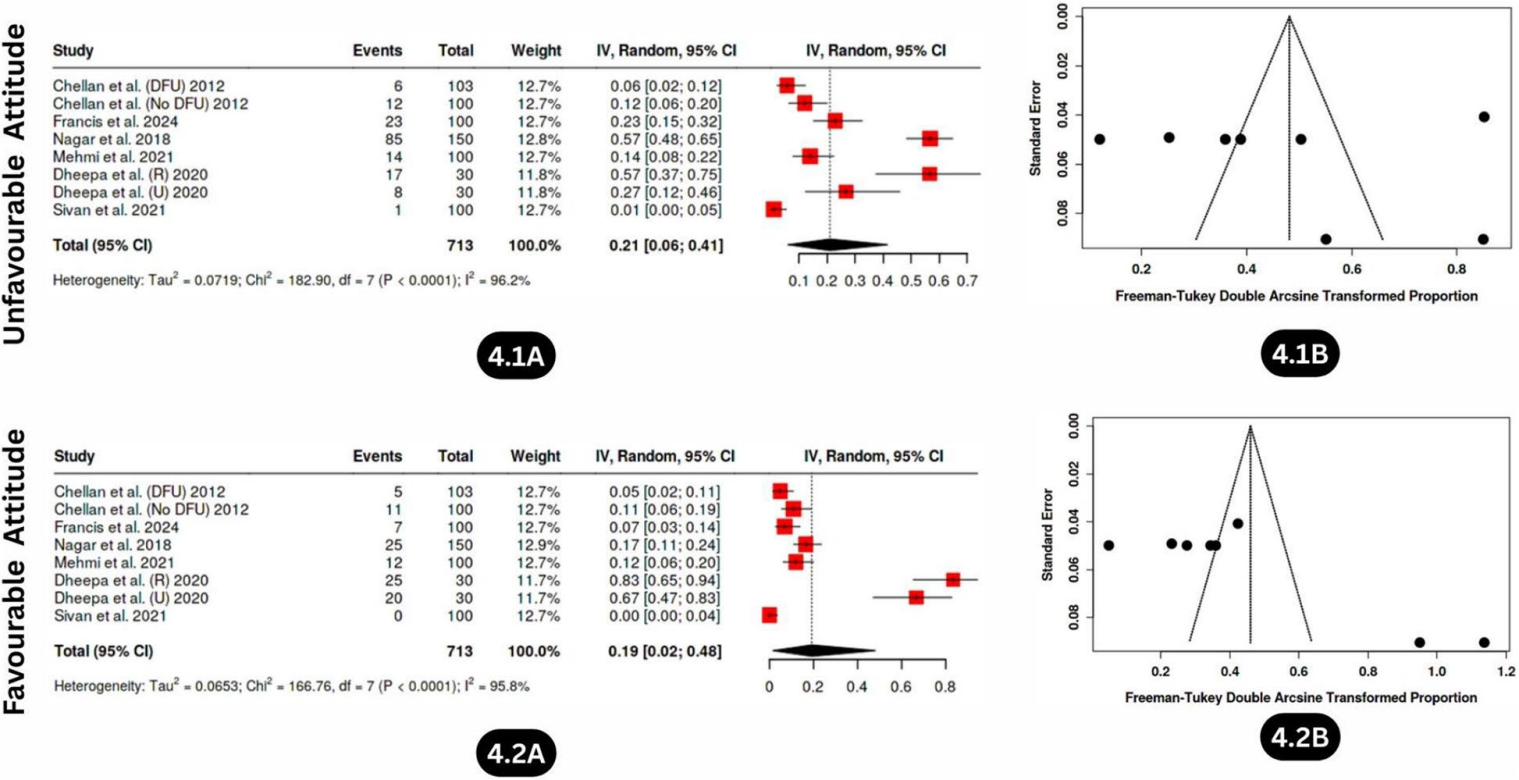
Forest plot and Funnel plot depicting the pooled estimates, publication bias of unfavourable attitude (4.1A, 4.1B), and favourable attitude (4.2A, 4.2B) levels related to diabetic foot care among individuals with diabetes in India

#### Favourable attitude

Analysis of six studies estimated that only 20% (95% CI: 2.0%–48.0%) of individuals demonstrated favourable attitudes toward diabetic foot care (Fig. 4.2A), indicating a widespread lack of motivation or interest in foot care. This estimate was accompanied by pronounced heterogeneity (I^2^ = 96%, *p* < 0.01), reflecting substantial variability across studies. Although publication bias was explored using funnel plot inspection and statistical tests (Egger’s intercept = 14.49, 95% CI: 4.28–24.69; t = 2.783; *p* = 0.032), these analyses were conducted on an exploratory basis due to the limited number of included studies and were interpreted with caution. The observed funnel plot asymmetry (Fig. 4.2B) may indicate small-study effects rather than definitive evidence of publication bias.

### Practice domain

#### Poor practices

Fourteen studies involving 2157 individuals with or without DFU and individuals with diabetes from both rural and urban areas were included in the meta-analysis, among which 36% of individuals reported poor foot care practices (95% CI: 23.0–50.0%) (Fig. 4.1A), with significant heterogeneity (I^2^ = 98%, *p* < 0.01), and no substantial publication bias was observed *(*intercept: 4.92, 95% CI: −3.47–13.31, t: 1.149, p value: 0.27) (Fig. 4.1B).

#### Satisfactory practices

Fourteen studies with 2,712 participants and only with or without the presence of DFU and individuals with diabetes were included in the meta-analysis; 45% of the studies reported satisfactory foot care behaviours (95% CI: 31.0%−60.0%) (Fig. 5.2A), with high heterogeneity (I^2^ = 98%, *p* < 0.01) and no publication bias (intercept: 6.69, 95% CI:−2.7–16.08, t: 1.397, *p* value: 0.188) (Fig. 5.2B).

**Fig. 5:**
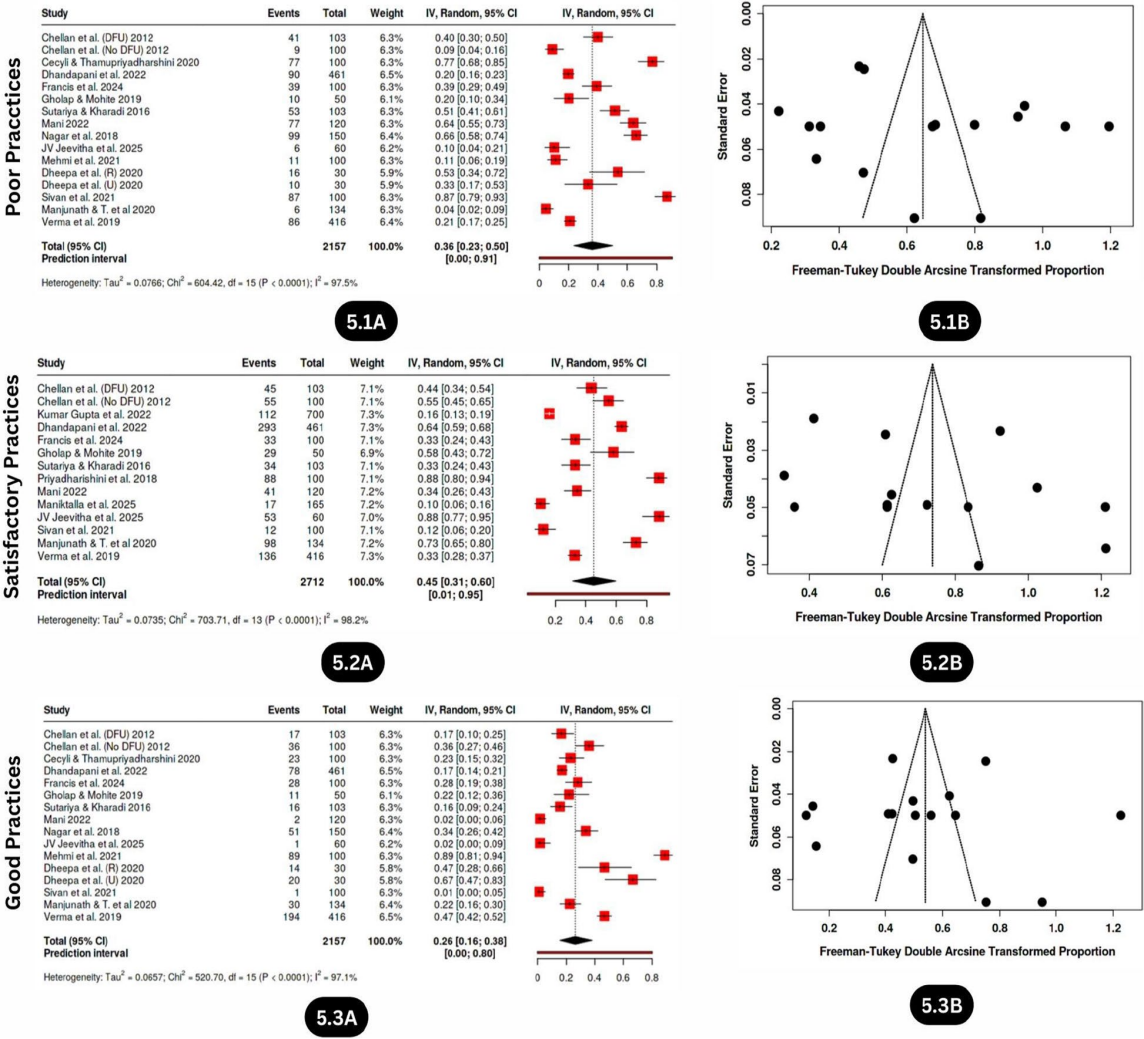
Forest plot and Funnel plot depicting the pooled estimates, publication bias of poor practice (5.1A, 5.1B), satisfactory practice (5.2A, 5.2B), and good practice (5.3A, 5.3B) levels related to diabetic foot care among individuals with diabetes in India

#### Good practices

Fourteen studies with 2157 individuals were examined for the pooled prevalence of good diabetic foot care practices, among which 26% (95% CI: 16.0%–38.0%) of individuals reported good foot care practices/behaviours (Fig. 5.3A), with high heterogeneity (I^2^ = 97%, *p* < 0.01) and no publication bias (intercept: −0.57, 95% CI:−8.71–7.56, t: −0.138, *p* value: 0.892) (Fig. 5.3B).

## Discussion

The findings of this meta-analysis collectively suggest that knowledge, attitudes, and practices (KAPs) related to diabetic foot care remain suboptimal among the studied population in India. This pattern indicates that while awareness of diabetic foot complications exists to some degree, significant gaps persist across all three dimensions, i.e., knowledge, attitudes, and practices, limiting the effectiveness of preventive and management strategies.

To enhance impact, these gaps can be addressed through a prioritized, stepwise intervention framework that sequentially targets deficits in knowledge, attitudes, and practices, while being supported by interprofessional and system-level strategies.

### Addressing knowledge gaps through contextualised education

The pooled prevalence of poor knowledge (34%) highlights a substantial awareness gap, with more than one-third of individuals lacking an essential understanding of preventive foot care. Similarly, only 32% of the participants reported good knowledge about foot care practices (95% CI: 18%–47%), whereas 34% reported poor knowledge (95% CI: 19%–51%), indicating a generally low level of awareness. These findings underscore the inadequacy of current health education efforts across different regions in India. Studies conducted in Kolkata [61] and Pondicherry [62, 63] revealed that many patients remain unaware of critical complications such as foot ulcers and sensory loss, reinforcing the urgent need for localized and accessible educational initiatives [61–63].

The variation in knowledge levels across studies may reflect differences in socioeconomic background, education, and healthcare access [17, 64]. In particular, rural and low-income populations often have limited exposure to foot care information, further widening the awareness gap [65–68]. On the positive side, structured teaching programs (STPs) [69] and community-based educational interventions, such as those implemented in Western Australian populations [70], have demonstrated marked success in improving knowledge. A study from Nagaland in Northeast India [69] reported significant gains in diabetic foot care knowledge after the implementation of STPs [69]. Similarly, postintervention studies reported better foot care knowledge and practices, leading to improved outcomes and reduced complications [71, 72].

Thus, the evidence suggests that standardized, culturally adapted [73], and visually engaging education [74] supported by periodic training and mobile health platforms [74] can effectively strengthen awareness, promote continuous learning, and empower patients in managing their foot health.

### Transforming knowledge into positive attitudes through behaviour-focused interventions

Despite educational progress, the translation of knowledge into positive attitudes remains limited [75, 76]. The pooled estimates for unfavourable attitudes (21%) and favourable attitudes (20%) indicate that awareness does not always lead to motivation or behavioural intentions. Only 20% of the participants exhibited favourable attitudes (95% CI: 8%–35%), reflecting the persistence of psychological and cultural barriers to proactive engagement in foot care. Stigma, fear, lack of motivation, and poor integration of foot care within healthcare systems are among the major factors influencing attitudes [3, 11, 64]. In addition, traditional habits such as barefoot walking and the shortage of trained healthcare providers continue to exacerbate this issue [3, 11, 64].

These findings indicate that educational interventions must be explicitly designed to influence beliefs, emotions, and perceived self-efficacy, rather than focusing solely on information delivery.

This highlights the need for behaviour-change-focused educational strategies that go beyond knowledge transmission [76]. The evidence suggests that motivational interviewing and family-based counselling approaches are more effective in reshaping patient perspectives and fostering adherence to preventive care [63, 69]. Addressing these attitudinal barriers requires interventions that integrate emotional support, myth correction, and culturally sensitive storytelling [77, 78], whereas patient narratives, local campaigns, and relatable role models [78, 79] can reinforce a sense of responsibility and self-efficacy.

### Enabling and sustaining preventive foot care practices

Compared with knowledge and attitudes, actual foot care practices reveal an even greater shortfall. The current meta-analysis revealed that 26% (95% CI: 11%–44%) of participants reported good practices, 45% (95% CI: 25%–66%) demonstrated satisfactory practices, and 36% (95% CI: 21%–52%) still engaged in poor practices. These findings highlight that knowledge and motivation alone do not guarantee consistent preventive action. This reflects not only a lack of awareness but also systemic obstacles such as limited access to screening, unaffordability of protective footwear, and inadequate training among healthcare providers [3, 11, 64]. Although hands-on training, follow-up support, and cost-effective care models have shown potential benefits [63], their implementation has often been fragmented and inconsistent across regions.

To bridge this gap, educational initiatives must be complemented by practical and structural measures, including hands-on demonstrations, subsidized footwear, and routine follow-ups to reinforce adherence. Encouragingly, the satisfactory practice level (45%) suggests that behaviour can be improved with sustained engagement. Mobile reminders [80, 81], behaviour-tracking applications [80, 82, 83], and caregiver involvement [75, 84] have been identified as useful tools to support daily self-care adherence.

However, the finding that only one-fourth of participants consistently follow recommended practices remains concerning. Strengthening diabetic foot care protocols within primary care, integrating regular foot assessments, and offering financial or insurance-based support for preventive care are crucial to improving long-term compliance and outcomes.

### Cross-cutting strategy: integrating interprofessional education and system-level support

Taken together, these results reveal a progressive decline from knowledge to practice, confirming that while awareness is present, its translation into sustained behavioural change remains weak. The high heterogeneity across studies (I^2^ > 75%) further suggests variability in design, population, and healthcare infrastructure, making outcomes difficult to generalize or sustain. Therefore, a more coordinated and collaborative framework is needed, one that combines education, motivation, and practical facilitation in an integrated, patient-centred approach.

In this context, interprofessional education (IPE) and interprofessional practices (IPP) have emerged as highly relevant strategies. Unlike traditional single-provider models, IPE & IPP bring together physicians, nurses, podiatrists, diabetes educators, physiotherapists, and community health workers to learn with, from, and about each other [85, 86]. This collaborative learning promotes shared responsibility, consistent patient messaging, and mutual reinforcement of preventive practices [87]. By fostering teamwork and cross-disciplinary communication, IPE & IPP enhances patient understanding, shapes positive attitudes, and standardizes preventive care, transforming fragmented efforts into sustainable outcomes.

Building upon the interprofessional (IP) framework, enhancing diabetic foot care requires action at multiple levels. At the patient level, accessible educational materials, community outreach to discourage improper foot care practices such as barefoot walking, and the reinforcement of preventive behaviours by healthcare providers remain essential [88, 89]. At the clinical level, multidisciplinary care models comprising podiatrists, nurses, physicians, and diabetes educators offer a holistic approach that has been shown to reduce amputation rates and improve outcomes [88, 90, 91]. Establishing specialized foot clinics, maintaining consistent follow-up, and adopting new tools such as skin temperature sensors can further aid early detection and prevention [90, 92–94].

Technology-driven interventions, especially telemedicine, have shown promise in bridging gaps in rural and underserved regions by facilitating early consultations and remote monitoring [95]. From a policy perspective, government support is vital for institutionalizing diabetic foot care. The development of national guidelines, equitable access to services, and the establishment of dedicated foot care centres must be prioritized [96–98]. Public health campaigns through media and community networks can help normalize preventive foot care behaviours [99].

Finally, the continuous training of healthcare professionals remains central to sustaining progress. Continuous medical education, interprofessional collaboration (IPC), and codesigned care protocols can enhance consistency, expand workforce capacity, and align preventive care across all levels of the health system [91, 98, 100]. Together, these coordinated actions can align knowledge, attitudes, and practices into a cohesive continuum, fostering sustainable improvements in diabetic foot health and reducing long-term complications.

### Limitations

Most of the included research originated from the southern and western regions of India, resulting in limited representation from the northeastern, central, and eastern states. This severe geographical underrepresentation, particularly of socio-economically and culturally distinct regions such as Bihar, Central India, and the Northeast, limits the national generalizability of the findings, and the pooled estimates should therefore be interpreted as region-specific rather than nationally representative. Significant heterogeneity among studies (I^2^ > 75%) likely stemmed not only from differences in methodology, tools, and population characteristics, but also from unmeasured regional disparities in socio-cultural contexts, economic conditions, and healthcare access that were insufficiently captured due to uneven geographical coverage. The predominance of cross-sectional designs limits causal inference. Moreover, cultural and linguistic diversity was not thoroughly considered. Incomplete demographic reporting in several studies further constrained deeper analysis of influencing factors.

## Conclusion

This systematic review reveals low levels of good knowledge (32%), favourable attitudes (20%), and effective practices (26%) concerning diabetic foot care among individuals with diabetes in India, with most studies concentrated in a few states and leaving large regions underrepresented despite their cultural and healthcare diversity. The findings emphasize that existing individual- or community-level educational interventions, although beneficial, remain insufficient, possibly owing to systemic barriers such as socioeconomic disparities, limited resources, and fragmented care delivery. Addressing these challenges requires a coordinated national strategy that prioritizes diabetic foot care education as a public health imperative. Culturally tailored educational programs, community health worker engagement, multilingual resources, a multidisciplinary approach, technology-enabled care, and supportive government policies may create a more equitable and sustainable model for diabetic foot self-management.

### Future directions

Future research should prioritize large-scale, nationally representative studies to capture regional disparities and cultural variations in KAP for diabetic foot care. Evaluating community-based interventions and technology-driven platforms, such as mobile health applications and telemedicine, may optimize preventive strategies and extend care to underserved populations. The incorporation of longitudinal study designs and patient-centred approaches has the capacity to generate stronger evidence of sustained behaviour change and long-term clinical outcomes.

## Sustainable development goals (SDGs)

This study aligns with key sustainable development goals (SDGs) related to health, education, and equality. It supports SDG 3: good health and well-being, particularly Targets 3.4, 3.8, and 3.c, by promoting early prevention and effective management of diabetic foot complications; addressing gaps in knowledge, attitudes, and practices (KAP); and strengthening healthcare workforce capacity through interprofessional education. It also contributes to SDG 4: Quality Education (Target 4.7) by advancing patient health literacy and fostering collaborative learning among healthcare professionals to encourage sustainable self-care practices. In line with India’s National Programme for Prevention and Control of Cancer, Diabetes, Cardiovascular Diseases and Stroke (NPCDCS)**,** this study emphasizes the need to integrate structured diabetic foot care and education-based interventions within primary healthcare systems to reduce complications and improve outcomes.

## Supplementary Information

Supplementary Material 1.

Supplementary Material 2.

## Data Availability

All data generated or analysed during this study are included in the tables and figures provided in the supplementary materials of the published article.
